# Combined Dorsal and Ventral Onlay Buccal Graft Technique for Large and Complex Penile Strictures

**DOI:** 10.1155/2018/1846060

**Published:** 2018-02-18

**Authors:** Nikolaos Mertziotis, Andreas Konandreas, Christos Kyratsas

**Affiliations:** IASO General Hospital, Athens, Greece

## Abstract

**Purpose:**

To present a modified technique of managing extensive penile urethral strictures with dorsal and ventral onlay buccal mucosa grafts.

**Patients and Methods:**

From October 2014 to January 2016, a total of 12 patients underwent urethroplasty for penile urethral strictures, using dorsal and ventral onlay grafts from buccal mucosa. The mean age was 42.75 (17–71). All patients completed the IPSS and QoL questionnaire, and uroflowmetry was done preoperatively. After surgery, the follow-up included completion of IPSS and QoL questionnaire and measuring of uroflow at 1, 3, 6, and 12 months. Postoperative urethrography was performed in complex cases or in the event of deterioration of voiding symptoms.

**Results:**

The mean length of the strictures was 5.45 (2, 2–16) cm. Mean Qmax changed from 3.45 ml/sec preoperatively to 18.33 postoperatively, and mean IPS score significantly decreased from 20.1 preoperatively to 8.98 postoperatively. All values were statistically significant (*p* < 0.001). No intraoperative or immediate postoperative complications were recorded. Overall, at 12 months, 11 out of 12 patients (91.6%) had a marked improvement in quality of life and uroflowmetry parameters.

**Conclusions:**

In the properly selected patient, the combined use of double graft for penile urethral strictures can be successful with minimal morbidity, at short-term follow-up.

## 1. Introduction

Urethroplasty is the treatment of choice for penile and bulbar urethral strictures, offering the best long-term results [1]. Although anastomotic urethroplasty is widely used for strictures up to 2 cm in length, more extensive strictures usually require some kind of graft augmentation, usually from buccal mucosa [2, 3]. Very often, the placement of a single graft, either ventrally or dorsally, is not sufficient to achieve adequate urethral caliber. The concurrent use of two grafts, on both the dorsal and ventral urethra (the “Palminteri” technique) has been proposed for extensive bulbar urethral strictures, with very satisfactory results [4]. Up to now, this technique has not been extensively studied for penile urethral strictures. Such strictures usually develop in men as a result of iatrogenic trauma [5]. Before the double graft, guidelines recommended a two-stage urethroplasty to achieve better results and reduce the possibility of graft rejection [6, 7]. The use of double graft could be a good alternative, as it could reduce the time to recovery of such patients. The aim of this study is to present the use of a modified Palminteri technique for patients with complex and/or extensive penile urethral strictures in a single procedure.

## 2. Materials and Methods

From October 2014 until January 2016, 12 patients aged 17–71 years (mean age 42.75 years) underwent urethroplasty with the use and application of free autologous graft on the dorsal and ventral surface of the urethra in one step by the same surgeon.

Preoperatively, all patients, besides the detailed history and physical examination, completed the IPSS questionnaire including the quality of life (QoL) domain and were evaluated with urine flow measurement, urethrogram (retrograde and during urination), and in some cases endoscopy and transabdominal ultrasound, in order to determine the nature and length of the stricture. 4 patients had a suprapubic catheter, while 6 of them had already been treated unsuccessfully for strictures caused by traffic accidents or correction of congenital hypospadias. 4 strictures were posttraumatic, 3 were iatrogenic, 2 were caused by lichen sclerosus (former balanitis xerotica obliterans), 2 were related to congenital hypospadias while 1 case was considered idiopathic. 2 patients had concurrent penile and bulbar urethral strictures.

### 2.1. Surgical Technique

Patients were advised to start mouth washouts with hexetidine solution 5 days before the operation. Preoperative antibiotics were usually used in the form of a fluoroquinolone 1 hour before the operation. Skin shaving of the scrotum and perineum was performed in the operating theater to reduce infection risk. General anesthesia was administered. Mouth exposure was achieved with the use of the Kilner–Doughty retractor, and the harvesting of the graft was always performed first. We prefer to use a single wide graft (from 6 to 10 centimeters long and 1.5 centimeters wide) which we then divide in two longitudinal halves, instead of harvesting from both cheeks. Oral mucosa was closed with a running 3–0 monocryl suture.

**Figure 1:**
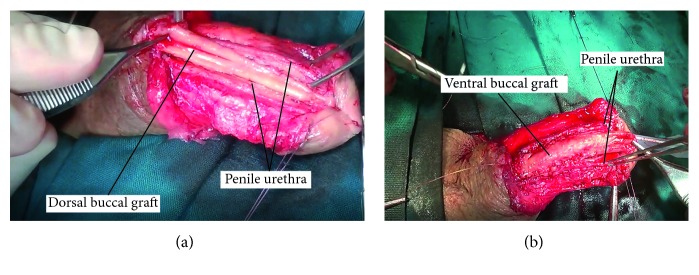
(a) Suturing the dorsal buccal graft on the tunica albuginea. (b) Placement and suturing in the urethra of the ventral buccal graft.

The rest of the procedure was usually performed with the patient in the supine position. A longitudinal penile skin incision was used to access the urethra, which was then mobilized with careful dissection through the dartos and Buck's fascia. A 16 or 18 Fr Nelaton catheter helped to identify the distal margin of the stricture. A longitudinal incision was then performed over the stenotic part of the ventral penile urethra and was extended up to the proximal margin of the stricture. A second incision was also performed on the dorsal surface of the urethral plate, involving the strictured area at its full length. Half strip of buccal graft was then sutured in dorsal inlay according to Asopa technique while the other half strip of the graft was placed as a ventral onlay (Figure 1). The corpus spongiosum, subcutaneous tissue, and skin were then closed with interrupted sutures. Care was taken to develop at least three subcutaneous layers without suture lines overlapping in order to minimize the risk of fistula formation.

The use of antibiotics was resumed immediately following the operation; patients were advised to have cold drinks and/or ice cream, as well as to follow a soft diet. Patients usually stayed in hospital for 48–72 hours. They completed a 10-day antibiotics course or continued taking antibiotics until the removal of the catheter, which was planned in 3 weeks postoperatively.

Follow-up included uroflowmetry at 1, 3, 6, and 12 months and urethrogram (when recurrence was suspected) and completion of the IPSS questionnaire. Surgical correction was considered successful when there was statistically significant improvement in Qmax, IPSS, and QoL score. The study also recorded operative time, catheterization period, and duration of hospitalization.

Statistical analysis was performed with the Stata MP Program 10.1® (StataCorp LP, Texas, USA). The regularity was tested using the Shapiro–Wilk test. The comparison with the Wilcoxon rank-sum test and the *t*-test was used for abnormal and normal distribution of values, respectively. Statistical significance was defined as *p* < 0.05.

## 3. Results

The mean stricture length was 5.45 cm (2.2–16) while the average follow-up was 8.91 months (3–14). The mean preoperative IPS score and QoL were 20.1 (9–32) and 3.26 (2–6), respectively, while mean preoperative Qmax was 3.45 ml/sec.

Postoperatively, the mean values for IPSS, QoL, and Qmax were 8.98, 1.35, and 18.33 ml/sec, respectively. The IPSS, QoL, and Qmax difference was −56.45%, −66.75%, and 158.16%, respectively, which was statistically significant for all three parameters (*p* < 0.001) (Table 1). Mean operative time was 186.25 minutes (90–270), mean duration of catheterization was 24.06 days (21–31), and mean hospitalization time was 2.81 days (2–4.5). The graft was harvested from buccal mucosa in 10 patients and from the penile foreskin in 1 patient, while in 1 patient there was a combination of both, because of extensive stenosis.

There were no intraoperative or immediate postoperative complications (Table 2), but only late adverse events. 2 out of the 4 patients with suprapubic catheter suffered from urinary tract infection (16.7%) after catheter removal (Grade II complication, according to the Clavien–Dindo classification system of surgical complications). Only 1 patient (8.3%) with a complex and extensive penile and bulbar urethral stricture (∼16 cm) developed a bulbar urethral stenosis in the anastomosis area with the membranous urethra, and he is currently managed by self-dilation (Grade IIIa complication, according to the Clavien–Dindo classification of surgical complications) [8]. This particular patient was one of four with suprapubic catheter due to urinary retention preoperatively. None of the patients developed a fistula or needed a blood transfusion.

## 4. Discussion

The use of double buccal mucosa grafts on the ventral and dorsal bulbar urethra, first described by Palminteri in 2008, since then had been applied only in small series to penile urethral strictures [4]. Our department has used the Palminteri technique with slight modifications (mainly as regarding the graft thickness) for penile urethral strictures. EAU Guidelines recommend a two-stage urethroplasty for better long-term results [6, 7]. We used not only buccal mucosa grafts but preputial grafts as well, in a single procedure. Although this is a retrospective study with few patients and a short follow-up, the technique looks very promising as it can provide adequate width of the urethra, and it can reduce the recurrence rate to minimum, even in complicated cases. The single patient who developed a recurrence had the longest stricture length, involving the largest part of the urethra, which was the result of a previous transurethral prostatectomy. The recurrence occurred in the area of the anastomosis of the graft with the membranous but not with the penile urethra.

One of our major concerns was the meticulous dissection through the scrotal layers of dartos and Buck's fascia so as to prevent the formation of urethrocutaneous fistula [9]. As a result, we did not experience any fistulae in our patients.

We regard the urinary tract infections that two patients developed as a result of the previously indwelling suprapubic catheters and the many courses of antibiotics that they had taken.

The high success rate of our series and the statistically significant improvement of uroflowmetry and IPS score are in concordance with the improvement in their quality of life scores, even in the patient who developed the restricture and eventually had to self-dilate every second week. This was not unexpected, given that this patient (who was the eldest in our series) had a long-term suprapubic catheter before the operation.

In conclusion, the concurrent use of double buccal mucosa from a single graft or preputial grafts in complex penile urethral strictures is a new technique, with excellent results as regards voiding parameters and quality of life. It can also reduce the recurrence and fistula formation rates common to older techniques. The outcome from its use in penile urethral strictures is encouraging but more extensive, prospective studies with larger series of patients and longer follow-up are needed.

## Figures and Tables

**Table 1:** Presentation of results.

Parameter	Mean		Difference (%)	*p* value
	Preoperative	Postoperative		
QoL	3.26	1.35	56.45	0.001
Qmax (ml/sec)	3.45	18.33	66.75	0.001
IPSS	20.1	8.98	158.16	0.001

**Table 2:** Presentation of complications.

Complications	Number patients	Percentage (%)	Clavien–Dindo classification
Fistula	0	0	—
Blood transfusion	0	0	—
UTI	2	16.7	II
Recurrence	1	8.3	IIIa
